# Editorial: Digital leadership: Competencies, business models, systems, strategies and platforms

**DOI:** 10.3389/fpsyg.2023.1137894

**Published:** 2023-02-06

**Authors:** Yashar Salamzadeh, Pelin Vardarlier, Ai Ping Teoh

**Affiliations:** ^1^School of Business and Management, University of Sunderland, Sunderland, United Kingdom; ^2^School of Business and Management Science, Istanbul Medipol University, Istanbul, Turkey; ^3^Graduate School of Business, Universiti Sains Malaysia, Penang, Malaysia

**Keywords:** digital leadership, digital transformation, digital platform, digital strategies, IR 4.0

As many organizations are now encountering the changes and transformations related to industrial revolution 4.0 (IR 4.0), they need to have a better view about their vision, and they do not need to limit this transformation to only technological changes such as application of artificial intelligence, big data, business intelligence, analytics tools, internet of things and so on (Vardarlier and Ozsahin, 2021). There are some other aspects of the organizations that are transforming as well and in many cases their impact is even more critical on them. Digital leadership is one of these emerging organizational concepts (Jagadisen et al., 2022).

After a decade of rapid technological growth, it has lately been discovered that many of these technologies have failed to take the human spirit into account to the extent that it deserves. As a result, a new movement has emerged to emphasize the role and significance of humans, as well as their sentiments and emotions. As a result, numerous practitioners and academics have launched new projects and efforts in this area, and the human-centric digital transformation is becoming more daring by the day (Khaw et al., 2022). We've recently heard a lot about the intersection between IR 4.0 and society 5.0, which is another new trend that emphasizes the influence of digital transformation on societies and people (Salamzadeh, 2022).

While studying, analyzing and designing policies for digital leadership, one of the initial steps is to know more about the digital competencies needed by the managers and their employees (Maruthuvellu et al., 2022). These digital leadership competencies facilitate the process of leadership from different stakeholders' perspective while organizations either use digital technologies or working in a digital business ecosystem. Having the wide range of skills and competencies under the big umbrella of digital leadership competencies, is a survival factor for businesses in IR 4.0 era (Cheng Soon and Salamzadeh, 2020).

In some cases, the business models and the processes used for daily management of the organizations need a revision. In today's businesses, it is clear that traditional approaches for managing business processes are no longer adequate. Only with the use of technology have intricate business procedures in organizations become manageable (Karaboga and Vardarlier, 2020). Rapid developments in technology have also broadened our understanding and use of human resource management. In a digital world, organizational data and resource management is more systematic and easily accessible (Vardarlier, 2020). These changes in business models and business practices, result in huge changes in not only the organizational life of the employees and managers but also their social life. This is another consideration point about the importance of the digital transformation in business models and functional processes of the businesses (Zafer and Vardarlier, 2021).

In line with what shared above, the main goal of this Research Topic is to shed some light to different aspects of digital leadership as one of the main challenges about this concept is the high diversity in the perspectives related to digital leadership. Only by putting all different components of digital leadership such as digital transformation and digital platforms and business models together, we can reach to a better understanding about the digital journey of organizations, and we can use this knowledge to design new jobs, new systems, and new organizations for the emerging digital world/s. Same as other types of changes, if we do not ride the wave of digital transformation correctly, we will find ourselves beneath it and knowing how to manage this change, is not an option anymore. The main question is how fast and with which quality we plan to be there.

In this Research Topic you can read four papers, from which two are focusing on digital transformation and two on digital platforms, of course form different perspectives.

On the first paper, Marino-Romero et al., on a paper titled “*A study of the factors which influence digital transformation in Kibs companies*,” are focusing on finding a better definition for digital transformation using a qualitative. Their main focus is on building intra-organizational competitive advantage using these factors. They found that digital transformation is a multidimensional phenomenon and have proposed a conceptual framework for the term with the strategic requirements of the market, organizations, public institutions, and technological infrastructures of the professional sectors.

The second paper by Türk, titled “*Digital leadership role in developing business strategy suitable for digital transformation*” is done by doing 34 interviews with organizational leaders and they have analyzed the paper using MAXQDA. In this study the link between digital leadership and digital transformation is studies deeply and many practical findings are concluded and As a result, a perspective on how digital leadership can contribute to businesses that want to develop strategies suitable for the digital transformation process is presented.

The third paper, prepared by Li et al., titled “*Research on value co-creation mechanism of platform enterprises in digital innovation ecosystem: A case study on Haier HOPE platform in China*” is done Based on the event system theory and taking Haier's hope platform as a vertical case study, this paper deeply explores the research mechanism of value creation of platform enterprises in the digital innovation ecosystem, and reveals the role and impact of digital innovation ability, openness, and business innovation model on the process of co-creation.

The last paper of this Research Topic is written by Wang et al. and is titled “*Price competition and blockchain technology adoption strategies of agents on the digital platform*.” This research is based on two competing agents selling the same type of goods on the same platform. The paper discusses agents' blockchain technology application strategies in three scenarios, which are defined by whether agents choose to apply blockchain technology to improve consumer trust or not. The findings have important practical significance for promoting the application of blockchain technology and alleviating the problem of price information asymmetry in platform businesses.

## Author contributions

All authors listed have made a substantial, direct, and intellectual contribution to the work and approved it for publication.
